# A pure invasive cribriform carcinoma of the breast with bone metastasis if untreated for thirteen years: a case report and literature review

**DOI:** 10.1186/1477-7819-10-251

**Published:** 2012-11-20

**Authors:** Wei Zhang, Zhichun Lin, Tongxian Zhang, Fen Liu, Yun Niu

**Affiliations:** 1 Breast Pathology Department and Laboratory, Tianjin Medical University Cancer Institute and Hospital, Key Laboratory of Breast Cancer Prevention and Therapy, Tianjin Medical University, Ministry of Education, Key laboratory of Cancer Prevention and Therapy of Tianjin, West Huanhu Road, Ti Yuan Bei, Hexi District, Tianjin 300060, China; 2 Center for PET-CT, Pingjin Hospital, Logistics University of Chinese People’s Armed Police Forces, Chenglin Road, Tianjin, Hedong District, 300162, China

**Keywords:** Pure invasive cribriform carcinoma, Breast, Bone metastasis

## Abstract

We report a case of pure invasive cribriform carcinoma of the breast, which had been untreated for thirteen years, being found with bone metastasis at initial presentation, because distant metastasis is rarely found in this tumor. A fifty-nine-year-old postmenopausal woman presented with a large left breast mass. Although she had noticed a lump in a left breast thirteen years ago, she had not sought treatment. The tumor had enlarged gradually since from one year before and become ulcerated. The two enlarged axillary lymph nodes were also palpable. After two cycles of neoadjuvant chemotherapy, she underwent left radial mastectomy with a free skin graft. Emission computed tomography result has confirmed bone metastasis. The histological diagnosis of the tumor revealed the pure invasive cribriform carcinoma, since over than ninety percent of invasive tumor components showed a characteristic cribriform growth, and the remainder was tubular carcinoma. She has been well without evidence of tumor recurrence for seven years after surgery and several routine postoperative therapies. Although with favorable prognosis, pure invasive cribriform carcinoma is still possible to develop into the advanced (Stage four) breast cancer if untreated for a long time. However, the survival of this patient for free disease after several locoregional and systemic therapies maybe provide a supplement for invasive cribriform carcinoma’s excellent prognosis.

## Background

Invasive cribriform carcinoma (ICC) of the breast is characterized by the predominant cribriform growth pattern of its infiltrating component. The prognosis of ICC has been reported to be excellent, similar to tubular carcinoma (TC), sometimes even approaching or equaling that of the general population [1-4]. According to the World Health Organization (WHO) new definition [5], pure ICC is a type of carcinoma with infiltrating components presenting an almost entirely (> 90%) invasive cribriform pattern. The lesions show a predominantly cribriform arrangement. The remaining components limited to TC are also included in the category of ICC. Cases with components of some carcinoma type other than TC (< 50%) are regarded as mixed-type ICC. Pure ICC is very rare and usually associated with smaller tumor sizes, less lymph node metastasis involvement, lower tumor stages and better prognosis compared with mixed ICC [1,5]. In addition, distant metastasis of pure ICC has rarely been found till now. In this paper, we report an advanced pure ICC case of the breast that had been left untreated for a considerable amount of time, with distant metastases (bone).

## Case presentation

**Figure 1:**
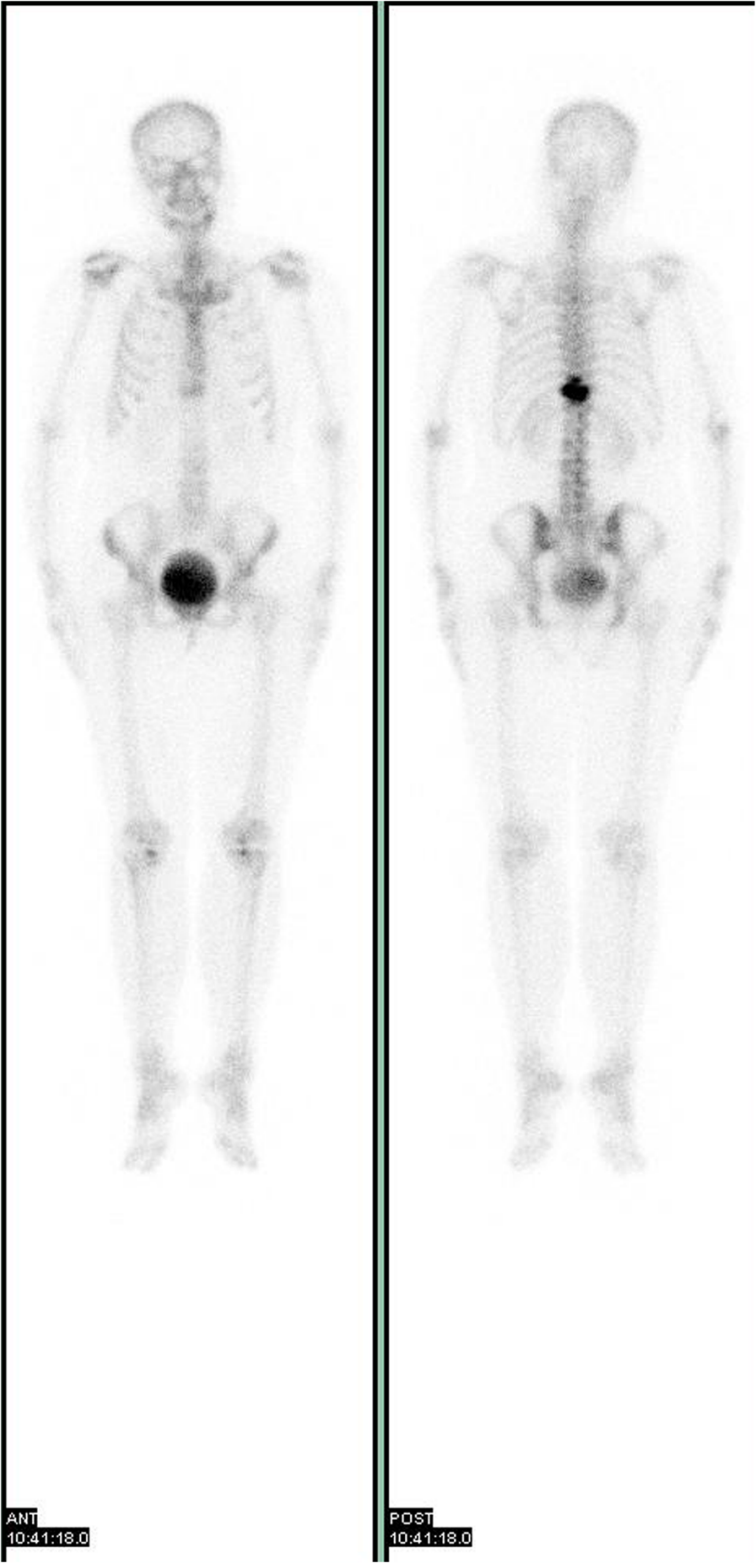
**The image of **^**99**^**Tc**^**m**^**-methylene diphosphonate (MDP) bone scintigraphy after left radical mastectomy.** The bone scan showed radioactive concentration in the tenth and eleventh thoracic vertebrae, indicating the appearance of bone metastasis at these sites.

A 59-year-old postmenopausal woman visited our hospital in November 2003 with a large left breast mass. She had noticed a lump approximately 2.0 cm in size in the superior external quadrant of the left breast in 1990 but did not consult a doctor. The tumor gradually grew and became ulcerated in 2002. Physical examination revealed a large mass (10 cm × 10 cm × 5 cm) in the whole left breast, with a superficial ulcer of an area of 7 cm × 7.5 cm in the superior external quadrant. An area of 7 cm × 3 cm at the interior side of the left nipple adhered to the tumor, which was fixed to the chest wall. The left nipple was invaginated and fixed. Two enlarged lymph nodes (4 cm × 4 cm and 3.5 cm × 3 cm) were found in the patient’s left axilla; these nodes had fused together and could not move freely. No mass was palpable in the opposite breast or in the bilateral supraclavicular fossa. Mammography excluded a lesion of the opposite breast. Thoracic computed tomography (CT) showed that the large tumor occupied the left breast and enlarged lymph nodes were detected in the left axilla. Moreover, the image of multiple nodules in both lungs raised the suspicion of distant lung metastasis but there was no further evidence to confirm this. Emission computed tomography (ECT) indicated that the tenth and eleventh thoracic vertebrae had bone metastases (Figure 1). Abdominal ultrasonography revealed no metastatic lesions in the abdominal organs.

After two cycles of neoadjuvant chemotherapy (cyclophosphamide, doxorubicin and fluorouracil, CAF), the volumes of both tumor and axillary lymph nodes decreased and one of the lymph nodes became impalpable. The patient’s red blood cell (RBC) and white blood cell (WBC) concentrations decreased to 2.44 × 10^12^/L and 3.48 × 10^9^/L, respectively. Her hemoglobin concentration also decreased to 66 g/L, possibly due to the side effects of chemotherapy. The patient received adjuvant hemopoietic treatment until her WBC reached normal levels.

**Figure 2:**
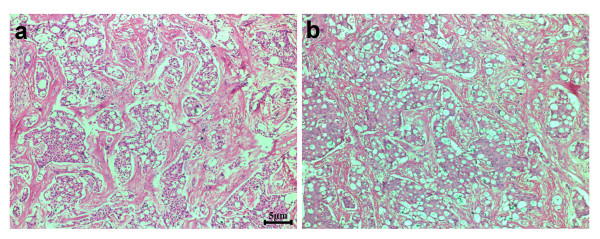
**Two representative areas show that most infiltrating tumor cells are arranged in groups with a cribriform growth pattern, with obvious sclerotic stroma between the tumor nests.** Scale bars = 5 μm in **a** and **b.**

**Figure 3:**
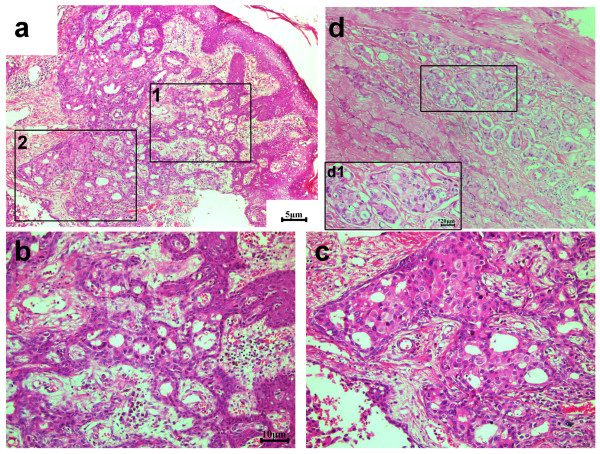
**Tumor cells invade into the skin and muscle.** (**a**) Numerous cribriform carcinoma cells present in the dermis of skin. Some tumor cells even infiltrate into the epidermis. Magnified views of box 1 and box 2 are displayed in (**b**) and (**c**), respectively. (**b**) The tumor cells invading the epidermis have grown in a cribriform pattern. (**c**) The invasive cribriform carcinoma may be the origin of ductal carcinoma *in situ*, often in a cribriform growth. (**d**) Some tumor cells invade the muscle. A magnified view is shown in the boxed area d1. The infiltrating carcinoma cells usually display in a cribriform pattern. Scale bars = 5 μm in **a** and **d**, 10 μm in **b** and **c**, and 20 μm in **d1.**

**Figure 4:**
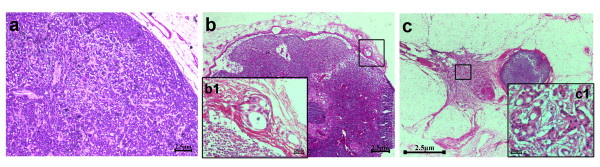
**The histology of axillary lymph node metastasis in the patient.** (**a**) The obvious metastasis is detected in one lymph node and the infiltrating tumors cells appear in a cribriform pattern. (**b**) A micro-metastasis is detected in one lymph node. A magnified view is shown in the boxed area b1; it shows the micro-metastatic cells in a cribriform growth pattern. (**c**) One lymph node indicates the infiltration of paranodal tissue. A magnified view is shown in the boxed area c1, also showing the invasive cells in a cribriform style. Scale bars = 2.5 μm in **a-c**, 20 μm in **b1** and **c1.**

The patient then underwent left radical mastectomy with the aim of cytoreduction. The skin defect after mastectomy was repaired with a free skin graft from the abdominal wall. Histological pathology confirmed the tumor as pure ICC because > 90% of its infiltrating components exhibited a characteristic cribriform pattern (Figure 2). The tumor occupied almost the whole left breast except for the inferior intermediate quadrant. The nipple and areola of the left breast were also involved and tumor cells had infiltrated the skin and muscles (Figure 3). Of the twenty-five axillary lymph nodes sampled, the presence of obvious metastasis and micro-metastasis was found in one lymph node (Figure 4a and 4b); infiltration of paranodal tissue was found in another lymph node (Figure 4c). Immunohistochemical staining indicated that 15% of the tumor cells were positive for estrogen receptor (ER) and progestogen receptor (PR). Approximately 30% of the tumor cells displayed incomplete membranous staining for human epidermal growth factor receptor 2 (HER2, 1+). Approximately 10% and 20% of the tumor cells showed weak nuclear staining for Ki67 and p53, respectively.

After surgery, the patient was continuously treated with intravenous CAF, the dosage of which was decided based on the square of the patient’s body. Treatment began on postoperative day 1 and continued for two-week intervals for four cycles. The patient then received postoperative radiotherapy on the tenth and eleventh thoracic vertebrae because of the possibility of bone metastasis, taking pamidronate at the same time. The patient received hormonal therapy afterwards. Tamoxifen was used for three years after radiotherapy and then replaced with letrozole for another two years due to the patient’s inability to tolerate the side effects of tamoxifen. Follow-up was carried out at three-month intervals during the first year, every six months during the second year, and every 12 months thereafter. The medical work-up consisted of regular physical checkups, imaging tests, such as chest radiograph, bone scan, and/or ultrasound, and examination for recurrences, second primary breast cancers, or other metastatic diseases. The patient remained well without any signs of tumor recurrence even seven years after the operation.

## Discussion

ICC is a rare invasive breast carcinoma with an excellent prognosis. The incidence of ICC was reported to range from 0.3% to 3.5% [1,6-8]. While the WHO earlier definition (2003) and Page’s description classified ICC into classical and mixed subtypes [1,9], the WHO new edition (2012) seems to weaken the concept of the classical ICC [5]. In any case, both the old and new WHO definitions propose that pure ICC should be defined as breast carcinoma with infiltrating components presenting an almost entirely (>90%) invasive cribriform pattern. Venable [6] reported 62 cases of ICC from 1,087 patients with primary breast carcinoma and found a five-year survival rate of 93.1% for all ICC cases (all died of ICC with < 50% invasive cribiform pattern), and 100% survival for ICC with 50% to 99% invasive cribiform pattern (including pure ICC). Page *et al*. [1] reported 51 cases of ICC (35 classical and 16 mixed ICC) over an average follow-up time of 12.5 years. They found 10-year survival rates of 97% for classical ICC and 50% for mixed ICC. One patient with classical ICC died from a second and contralateral carcinoma, not the primary ICC. Ellis *et al*. [7] compared 10-year survival rates for different histological types in 1,621 cases of primary breast cancer. Ductal carcinoma *in situ*, cribriform and TC had excellent prognoses, with 10-year survival rates of 92%, 91% and 90%, respectively, compared with 80% for mucinous carcinoma, 51% for medullary carcinoma, 54% for lobular carcinoma and 47% for invasive ductal carcinoma-not other specified (IDC-NOS). The frequency of metastasis in the axillary lymph nodes was lower in pure ICC than in mixed ICC (14.3% vs. 25%, respectively) [1]. Venable found that the maximal number of positive lymph nodes in pure ICC was three [6]. To date, evidence of distant metastasis in ICC have been limited, especially for pure ICC.

In this paper, we report a case of pure ICC with bone matastasis that had been left untreated for 13 years. The patient’s pathology revealed that over 90% of the invasive tumor cells grew in a cribriform pattern, thus, the patient was considered to have pure ICC. The tumor cells that had locally invaded the nipple, skin and muscle were still arranged in a cribriform pattern (as indicated by Figure 3). Histology verified only one obvious metastatic lymph node and one micro-infiltrating lymph node, possibly due to the effect of two cycles of neoadjuvant chemotherapy received by the patient. Although we detected multiple nodules in both lungs during CT imaging on initial examination, we could not ascertain whether or not the patient had lung metastasis because she did not show any pulmonary symptoms at her first diagnosis and subsequent follow-up. Surprisingly, the patient was alive seven years after neoadjuvant chemotherapy, radical mastectomy, and postoperative systemic therapy. Taking the 13 years prior to treatment into account, this patient had survived for at least 20 years after the discovery of the lump in her breast.

Page [1] first reported that the average age of classical ICC is 53 years, younger than the age in mixed ICC (63 years). Venable reported that the mean age of patients with ICC is 58 years, including individuals whose tumors contain a cribriform pattern in less than 50% of cells [6]. Although the age at diagnosis of the patient in the present case was 59 years, her actual age during discovery of the lump was 46 years, younger than in the Page and Venable reports. Our recent retrospective investigation of 51 ICC cases found that the average age of patients with pure ICC was 51 years (unpublished data). We thus speculate that the different ages at diagnosis may contribute to this difference because breast carcinomas may now be detected earlier with the use of more advanced detection techniques and increased awareness of self-examination. Second, ethnic differences might result in this disparity, which was confirmed by recent publications on IDC-NOS [10-12]. The ICC tumor may present as a mass but is frequently clinically occult, and may escape radiological detection. Thus, lesions are usually larger at presentation, although they grow slowly over time [7].

The patient in this case noted a lump with a diameter of 2.0 cm 13 years ago but did not consult a doctor. The tumor finally grew to a large mass with a diameter of 10 cm. The nipple, skin, and muscle were also involved. The patient’s pathology revealed well-differentiated invasive tumor cells because they were arranged in irregular islands with extensive gland lumen formation. Cytologically, the nuclear grade is usually low or moderate, and the karyokinesis is rare. The remaining components of this tumor (< 10%) comprised TC, which has been confirmed to be occasionally associated with ICC (23%) [6]. Taylor and Norris [13] speculated that TC may, in some instances, serve as a differentiation bridge between intraductal carcinoma and ICC. Previous studies have indicated that in ICC there is expression of ER, but mostly there is expression of PR (69%), which could contribute to the favorable prognosis of this breast carcinoma [1,5,6]. ER and PR in this pure ICC case were found in 15% of the tumor cells even after 13 years of disease development. We thus speculate that the persistent expression of hormone receptors in pure ICC is a factor that influences the excellent prognosis of the carcinoma, given that the tumor at initial mass was positive for ER, since no publications of negative ER for pure ICC have been reported to date [1,5,6]. Previous publications [1,5-7] indicated that only metastatic lymph nodes in pure ICC are arranged in a cribriform pattern, similar to primary ICC tumors; such findings were confirmed by our results. Tumor cells in one micro-infiltrating lymph node and in other paranodal tissues also displayed a similar morphology (Figure 4b and 4c).

To date, the prognostic significance of the clinicopathological characteristics of ICC patients has not yet to be well established because of the low incidence rate and lack of a standard definition for the carcinoma. Thus, treatment guidelines for ICC are mostly extrapolated from data based on IDC without clear validation. The case we reported was a late-stage (T4) breast carcinoma for which comprehensive therapy was carried out. Neoadjuvant chemotherapy was performed because of the large size of the mass and the presence of two enlarged axillary lymph nodes. Radical corrective surgery for cytoreduction and endocrine therapy followed., Thoracic radiotherapy was also performed because of the possibility of bone metastasis. Several recent publications have questioned whether adjuvant chemotherapy is suitable in ICC cases, because reports on the efficacy of adjuvant or neoadjuvant chemotherapy in patients with ER-positive disease are limited [14-16]. Colleoni [4] recommended no therapy or endocrine therapy alone for favorable histotypes (such as, tubular, cribriform, mucinous, papillary) with luminal tumors. Caution must be exercised when making such a decision. Neoadjuvant chemotherapy was partially effective for this patient because of the shrink in both the tumor and axillary lymph node. However, we cannot exclude the roles that postoperative local and systemic therapies played in the patient’s excellent prognosis. We believe that definition of specific niches for tailored research through international cooperation and prospective investigations of this topic are necessary to make progress and achieve a consensus on the treatment of individual patients with ICC.

## Conclusions

Pure ICC is a rare and special histological form of breast cancer with excellent prognosis. At present, distant metastasis of pure ICC has rarely been reported inside or outside of the country. The current study presents a large left breast mass that had been untreated for 13 years and exhibited axillary lymph node and bone metastases. This mass was histologically verified to be pure ICC. Such a progression is not an ordinary development for pure ICC and surgeons should be aware that pure ICC may develop into advanced breast cancer when left untreated for a long period of time. The survival of the patient discussed in this study after several routine therapies indicates the excellent prognosis for pure ICC.

## Consent

Written informed consent was obtained from the patient for publication of this case report and any accompanying images.

## Abbreviations

CAF: Cyclophosphamide, doxorubicin and fluorouracil; CT: Computed tomography; ECT: Emission computed tomography; ER: Estrogen receptor; HER2: Human epidermal growth factor receptor 2; ICC: Invasive cribriform carcinoma; IDC-NOS: Invasive ductal carcinoma-not otherwise specified; PR: Progestogen receptor; RBC: Red blood cell; TC: Tubular carcinoma; WBC: White blood cell; WHO: World Health Organization.

## Competing interests

The authors declare that they have no competing interests.

## Authors’ contributions

ZW and NY conceived the study. ZW carried out the design, and collection of data and clinical records of the patient, and drafted the manuscript. LZC participated to collect the patient data and helped to explain the radiological information. ZTX and LF carried out the histopathological evaluation and collected the pathological images. NY participated in its design and helped revise the manuscript. All authors read and approved the final manuscript.
